# Morphology of Thin-Film Nafion on Carbon as an Analogue of Fuel Cell Catalyst Layers

**DOI:** 10.1021/acsami.3c14912

**Published:** 2024-01-11

**Authors:** Corey R. Randall, Lianfeng Zou, Howard Wang, Jingshu Hui, Joaquín Rodríguez-López, Melodie Chen-Glasser, Joseph A. Dura, Steven C. DeCaluwe

**Affiliations:** † Colorado School of Mines, Golden, Colorado 80401, United States; ‡ NIST Center for Neutron Research, Gaithersburg, Maryland 20899, United States; § Clean Nano Energy Center, State Key Laboratory of Metastable Materials Science and Technology, Yanshan University, Qinhuangdao 066004, Hebei, China; ∥ University of Maryland, College Park, Maryland 20742, United States; ⊥ University of Illinois at Urbana-Champaign, Urbana, Illinois 61801, United States

**Keywords:** Nafion phase segregation, PEMFC, neutron reflectometry, catalyst layer, thin films

## Abstract

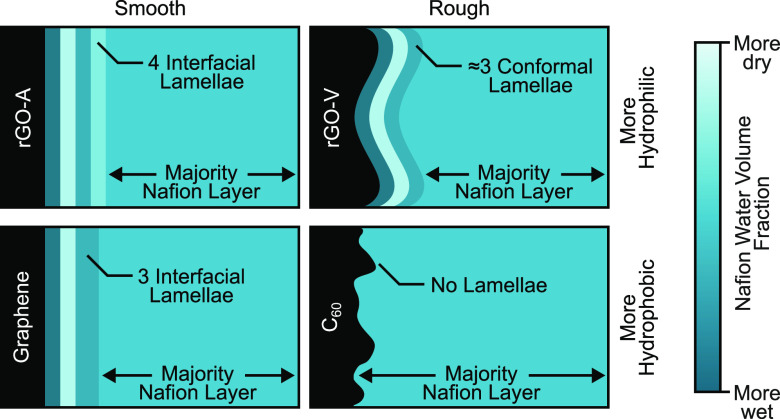

Species transport in thin-film Nafion heavily influences proton-exchange membrane (PEMFC) performance, particularly in low-platinum-loaded cells. Literature suggests that phase-segregated nanostructures in hydrated Nafion thin films can reduce species mobility and increase transport losses in cathode catalyst layers. However, these structures have primarily been observed at silicon–Nafion interfaces rather than at more relevant material (e.g., Pt and carbon black) interfaces. In this work, we use neutron reflectometry and X-ray photoelectron spectroscopy to investigate carbon-supported Nafion thin films. Measurements were taken in humidified environments for Nafion thin films (≈30–80 nm) on four different carbon substrates. Results show a variety of interfacial morphologies in carbon-supported Nafion. Differences in carbon samples’ roughness, surface chemistry, and hydrophilicity suggest that thin-film Nafion phase segregation is impacted by multiple substrate characteristics. For instance, hydrophilic substrates with smooth surfaces correlate with a high likelihood of lamellar phase segregation parallel to the substrate. When present, the lamellar structures are less pronounced than those observed at silicon oxide interfaces. Local oscillations in water volume fraction for the lamellae were less severe, and the lamellae were thinner and were not observed when the water was removed, all in contrast to Nafion–silicon interfaces. For hydrophobic and rough samples, phase segregation was more isotropic rather than lamellar. Results suggest that Nafion in PEMFC catalyst layers is less influenced by the interface compared with thin films on silicon. Despite this, our results demonstrate that neutron reflectometry measurements of silicon–Nafion interfaces are valuable for PEMFC performance predictions, as water uptake in the majority Nafion layers (i.e., the uniformly hydrated region beyond the lamellar region) trends similarly with thickness, regardless of support material.

## 1 Introduction

In the push to decarbonize the global energy infrastructure, proton-exchange membrane fuel cells (PEMFCs) have a promising role in applications requiring high gravimetric power density.^1,2^ PEMFCs convert chemical energy from hydrogen gas into electrical energy, producing only minimal greenhouse gas byproducts. Unfortunately, currently available PEMFCs are plagued by low durability, low performance, and high cost, preventing widespread adoption of the technology.^3−7^

PEMFC cost, durability, and performance concerns are not mutually exclusive. These issues largely stem from phenomena in the cathode catalyst layer (CCL), where transport and kinetic processes limit cell performance. Necessary electrochemical reactions occurring in the CCL require carbon (electron conductor), Nafion (proton conductor), Pt (catalyst), and nearby void space for gas-phase transport. At present, state-of-the-art PEMFCs used in fuel cell electric vehicles (FCEVs) require about 30 g of Pt per vehicle^8^—accounting for roughly half of the power-normalized stack cost.^9^ Attempts to lower PEMFC costs by reducing the Pt loading show performance losses beyond those expected from reduced catalyst surface area.^10−14^ At present, literature attributes these losses to species transport resistance in CCL Nafion.^9,15−17^

Nafion films in the CCL coat and connect Pt-decorated carbon particles and range in thickness from 5 to 20 nm. This is orders of magnitude thinner than the bulk Nafion electrolyte membranes (hundreds of μm) that separate PEMFC electrodes. Researchers have thoroughly explored bulk Nafion properties/morphologies and consistently report that interconnected water pathways develop uniformly and isotropically in hydrated bulk membranes.^18−23^ Furthermore, empirical relationships reveal that bulk Nafion’s ionic conductivity depends only on the relative humidity (RH) and temperature of its local environment.^24^ In contrast, experimental results from thin-film Nafion demonstrate notable differences in both structure and transport properties compared to bulk membranes,^25,26^ as described below.

Over the past 15 years, neutron reflectometry (NR) has provided new insights into the structure and water uptake of thin-film Nafion. NR is a broadly useful technique for determining the depth profiles of layered thin-film samples. The profiles can determine layer thicknesses greater than roughly 1.5 nm with sub-Ångstrom spatial resolution. Moreover, due to the high SLD contrast between Nafion and water, the technique is very sensitive to the location and amount of water in Nafion.^27^

Together, these capabilities provide the sensitivity to resolve spatial depth profiles in hydrated thin-film Nafion. Recent NR studies of hydrated Nafion thin films show that confinement impacts water uptake, which decreases with decreasing film thickness below roughly 60 nm.^28^ These studies conclude that humidified Nafion thin films can develop phase-segregated lamellae near substrate interfaces, i.e., in-plane water-rich and water-poor regions in the film.^17,28−31^ To derive predictive relationships for thin-film Nafion ionic conductivities, DeCaluwe et al. determined that both water uptake and structure are important.^28^ In addition to how much water the Nafion absorbs, it also matters where it is located, with reduced species mobilities in near-substrate Nafion, an idea supported by subsequent measurements from Farzin, et al.^32^ Despite the significance of these studies, most measurements have focused on Nafion at silicon interfaces, primarily due to the availability of smooth, polished Si wafers for model system measurements. However, in low-Pt-loaded PEMFCs, carbon–Nafion interfaces are dominant in the CCL, and silicon–Nafion interfaces are nonexistent, making it uncertain how applicable results from silicon–Nafion experiments are to PEMFCs.

Though less common, NR experiments with more complicated Pt–Nafion and carbon–Nafion interfaces demonstrate important differences in thin-film Nafion compared to silicon–Nafion.^29,33−36^ Whereas multilayered phase segregation is consistently observed in Nafion at silicon interfaces, Nafion near Pt is more likely to develop a single water-rich layer.^29,33,35^ Near carbon interfaces, a conclusive Nafion morphology remains elusive. Carbon black (CB) supports used in PEMFC CCLs consist of large particles with high surface roughnesses and therefore cannot be tested using NR, which requires nanometer-smooth samples. The few available carbon–Nafion NR publications therefore use carbons other than CB. Results demonstrate that interfacial Nafion structures depend on the type of carbon used.^33,34^ Although Nafion on CB cannot be directly tested using NR, thin-film Nafion morphologies in CCLs can be inferred by examining structures on a variety of carbon substrates with similar roughness, surface chemistry, and hydrophilicity to CB. Furthering our understanding of carbon–Nafion interfacial structures in CCLs provides a pathway toward a mechanistic explanation for reduced species transport in low-Pt-loaded PEMFCs.

In this study, we use NR and X-ray photoelectron spectroscopy (XPS) to thoroughly investigate carbon–Nafion interfaces. We fabricate and test Nafion thin films (≈30–80 nm) on four different carbon substrates, comparing our carbon samples to CB using XPS. The NR results reveal lamellar phase-segregated regions near some carbon interfaces and more isotropic phase segregation near others. When present, lamellar regions are less prominent or persistent than those observed near silicon interfaces. The variety of Nafion morphologies observed here demonstrates how thin-film structures are impacted by substrate interactions. Hydrophilic surfaces’ effects on phase-segregated structures were previously proposed by Dura et al.,^29^ and later observed on spin-on glasses by Kim et al.^31^ and on carbon surfaces by Ito et al.^34^ Here, we provide evidence that hydrophilicity is one of several characteristics impacting interfacial carbon–Nafion structures; another is surface roughness. Consequently, CCL Nafion on hydrophobic and rough CB supports is presumably less structured than Nafion on silicon. This conclusion suggests that reduced species mobilities and high transport losses in low-Pt-loaded PEMFCs are more a result of isotropically confined/restricted hydrated domains rather than lamellar phase segregation.

## 2 Experimental Methods

**Figure 1:**
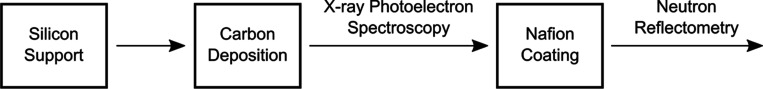
Generalization of the sample fabrication and the experiments performed in this study. Four samples were made, each with a different carbon-based layer deposited onto a silicon substrate. XPS characterized the surface chemistry of each carbon. Afterward, each carbon was coated with a Nafion thin film and NR measurements were taken.

Our process is generalized in Figure 1. In short, silicon supports act as a base for all samples. Using an assortment of deposition techniques, four different carbon layers were coated atop separate supports. XPS was used to characterize the carbon surfaces. Following these measurements, thin Nafion films were spin-coated atop the carbon films from a dilute Nafion solution. NR experiments using both dry and humidified sample environments were then performed. Humidified environments provide conditions similar to those found in the CCLs of operating PEMFCs. Experiments in dry conditions help when fitting reflectivity data, as explained in Section 3.2. Additional details of sample fabrication, XPS measurements, and NR experiments are available in the following subsections.

### 2.1 Sample Fabrication

NR experiments require samples with nanometer-smooth surfaces. Consequently, rough CBs commonly found in PEMFC CCLs (e.g., Vulcan XC-72 and Ketjen Black) are not viable. Therefore, Nafion films on four smooth-surface carbons were fabricated and tested. Each sample is made from the same basic elements: a polished silicon wafer (0.0762 m diameter), a carbon layer, and a Nafion thin film. Throughout this article, the name of the carbon layers differentiates samples. Two samples are based on reduced graphene oxide (rGO) and are referenced as “rGO-A” and “rGO-V”. The tailing letters (i.e., -A and -V) correspond to how these samples were annealed, as described below. The remaining samples are referenced as “graphene” and “C_60_.”

#### 2.1.1 rGO-A and rGO-V Depositions

A 1.25 mg mL^–1^ solution of dispersed graphene oxide in H_2_O (Sigma-Aldrich)^a^ was spin cast onto two separate silicon supports at 25 Hz (1500 rpm) for 60 s. Afterward, a hydrazine reduction was completed at 60 °C for 12 h. These two samples were annealed under separate conditions. The intention behind the separate annealing environments was to create distinct surface oxidations between the two rGOs. One sample was annealed in an argon environment (rGO-A) at 300 °C for 1 h. The second was annealed under vacuum (rGO-V) at 180 °C for 24 h.

#### 2.1.2 Graphene Growth and Transfer

The graphene layer was fabricated via chemical vapor deposition (CVD) and transferred to a silicon wafer through a wet-etching process using previously published procedures.^37,38^ In short, graphene was grown on a sacrificial copper catalyst (25 μm thick, purchased from Alfa Aesar) with 100 sccm of CH_4_ and 50 sccm of H_2_ as the gas source. The tube furnace was controlled at 1000 °C and 5 Pa during the 25 min deposition. After CVD growth, the fresh graphene surface was protected by spin-coated PMMA layers, and the bottom copper foil was removed with an etchant (CE-100, purchased from Transene Company). The thoroughly rinsed PMMA/graphene sheets were transferred onto target substrates and blow-dried using argon gas. The PMMA-protect layer was removed by immersing samples in anisole, a 1:1 DCM–acetone solution, and IPA.

#### 2.1.3 C_60_ Deposition

C_60_ was thermally evaporated onto a silicon wafer using an MBraun Thermal Evaporator at the National Renewable Energy Laboratory. The raw crystalline powder was purchased from Sigma-Aldrich. Roughly 8 nm of C_60_ was deposited onto the sample at a rate of 0.02 nm s^–1^. This is expected to be a nonepitaxial film, with a rough surface, more comparable to CB.^39^

#### 2.1.4 Thin-Film Nafion Coatings

A dispersion of 20 wt % Nafion 1100 in lower aliphatic alcohols and water (Sigma-Aldrich) was further diluted in HPLC-grade ethanol (Sigma-Aldrich). The mixture was sonicated for 20 min before being spun onto each sample at 58 Hz (3500 rpm) for 60 s with a Specialty Coating Systems 6800 Series spin coater. After spinning, samples were heated under vacuum at 60 °C for at least 1 h to remove solvent, promote surface adhesion, and ensure consistent thermal histories. A 1:16 volumetric ratio of Nafion to ethanol was used for the rGO-A, rGO-V, and graphene samples, resulting in ≈50–70 nm-thick films. The C_60_ sample was made at a later date using a 1:19 ratio to obtain a thinner film, ≈30 nm, that more adequately represents Nafion coatings in PEMFC CCLs (i.e., films <20 nm).

### 2.2 X-ray Photoelectron Spectroscopy

XPS is a nondestructive surface analysis technique that quantifies a material’s near-surface chemical composition. In this study, XPS measurements were taken to characterize the four carbon substrates (see Section 2.1). Data was used to determine similarities and differences between each carbon sample and CB, as discussed in Section 3.1. The equipment and operation for XPS tests are presented below.

Due to the timeline for testing and equipment availability, XPS was measured using three separate instruments. Survey scans were completed to quickly examine surfaces, check for impurities, and identify binding energies of interest for high-resolution scans. Observing no major impurities, high-resolution spectra were collected for the C 1s peaks. A minimum of two scan areas were examined for each sample to check for uniformity in the carbons’ surface chemistry.

XPS for the rGO-A and rGO-V samples was measured at Colorado State University (CSU) on a PHI Physical Electronics PE-5800 X-ray Photoelectron Spectrometer. On this instrument, Al Kα X-rays (1486.6 eV) at 350 W were used to scan large areas, roughly 3 × 3 mm^2^. Pass energies of 200 and 25 eV were used for the survey and high-resolution spectra, respectively. While the scans were performed, samples in the analysis chamber were held at a pressure below 3 × 10^–6^ Pa.

A Kratos Axis Ultra DLD instrument at the National Institute for Standards and Technology (NIST) was used to examine the graphene layer. During operation, the sample was kept under vacuum (4 × 10^–7^ Pa), while a 150 W Al Kα X-ray source (1486.6 eV) scanned 300 μm by 700 μm elliptical areas. Wide and high-resolution scans used 80 and 20 eV pass energies, respectively.

Measurements for C_60_ were taken on a Scienta-Omicron HiPP-3 system at the Colorado School of Mines (CSM). This instrument uses an Al Kα X-ray source (1486.6 eV) at 300 W. Each scan examined a 500 μm-diameter spot size on the C_60_ surface. As with the other tests, data collection was performed under vacuum (pressures <1 × 10^–4^ Pa). Survey and high-resolution spectra were collected using 200 and 100 eV pass energies, respectively.

### 2.3 Neutron Reflectometry

Specular NR measures the intensity of a reflected neutron beam as a function of the scattering vector *Q*_*z*_ (nm^–1^). The scattering vector is defined as
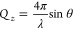
1where λ (nm) and θ are the wavelength of the neutron source and the grazing angle of the beam, respectively. Neutrons entering the sample interact with layered interfaces, leading to interference in the reflected neutrons, resulting in complicated intensity oscillations as a function of *Q*_*z*_, called Kiessig fringes. In short, this data is fit by proposing a 1D depth profile of the sample’s scattering length density (SLD, nm^–2^), simulating the reflectivity pattern from this profile, and adjusting the profile until the simulated reflectivity matches the measured data.

Fitting NR data therefore determines a 1D depth profile of a sample’s SLD in the through-plane direction. The SLD at each depth *z* (nm) is a linear combination of the SLDs and volume fractions *V_i_* for each phase *i* at that depth
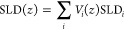
2

SLD profiles in this work supply an important insight into thin-film Nafion morphology and water uptake—both of which impact species transport in PEMFC CCLs.^28^

We conducted NR experiments at two facilities. The rGO-A, rGO-V, and graphene samples were measured using the Advanced Neutron Diffractometer/reflectometer (AND/R) and the Polarized Beam Reflectometer (PBR) at the NIST Center for Neutron Research (NCNR).^40^ The C_60_ sample was tested using the Liquids Reflectometer (LIQREF) at the Spallation Neutron Source (SNS) at Oak Ridge National Laboratory. Although instruments at both facilities perform NR experiments, their setup and operation differ. Details for each instrument are given below.

#### 2.3.1 NR Procedures for AND/R and PBR

The AND/R and its later evolution, MAGIK, and the PBR have been used in previous studies by some of the present authors.^17,28−31,36^ A detailed explanation for data collection on these instruments is given in refs (17), (27), and (40). Here, we provide only a brief summary.

A custom sample environment designed by NIST scientists is used to control the RH and temperature during NR experiments. The environment combines a dew point generator (DPG) and thermal management equipment to maintain user-specified conditions. Dry argon is humidified in the DPG by either H_2_O or D_2_O. Herein, all RH conditions are in reference to H_2_O-humidified flows. H_2_O (SLD = −0.56 × 10^–4^ nm^–2^) was used over D_2_O (SLD = 6.36 × 10^–4^ nm^–2^) for these measurements because it has a higher contrast with Nafion (SLD = 4.16 × 10^–4^ nm^–2^). Samples tested in this environment were held at 29.6 ± 0.2 °C in both dry (0% RH) and wet (90 ± 1.5% RH) conditions.

#### 2.3.2 NR Procedures for LIQREF

At the time of data collection, a sample environment capable of active RH control was not available at the LIQREF. Therefore, RH conditions were managed by placing samples inside a sealed aluminum can with beakers of either desiccant or saturated saltwater solutions. Measurements performed with desiccants simulate a dry environment by removing moisture from the air. Although the RH values of these tests were not measured, results refer to them as 0% RH. For the wet condition, a saturated solution of NaCl in water was placed in the sealed container. NaCl-saturated water maintains a RH of 75 ± 1% over a wide range of temperatures.^41^ Therefore, throughout the results, the wet condition for C_60_ is labeled as 75% RH. Tests on the LIQREF were conducted at room temperature, i.e., 21 ± 1 °C.

Aside from the sample environment, operation of the LIQREF also differs from that of AND/R in a couple significant ways. Rather than purely adjusting the angles of the sample and detector to vary *Q*_*z*_, the LIQREF uses a time-of-flight technique to vary the wavelength of the beam between 0.25 and 1.75 nm. With this, sample and detector positions only need to be adjusted 1–3 times to scan the same range of *Q*_*z*_ as on AND/R. For this study, the sample stage and detector were set to three angles: 2θ = 1.20, 2.37, and 4.69° for each C_60_ measurement. Additionally, the 2D position-sensitive detector on the LIQREF enables counting specular and off-specular reflectivity simultaneously. Therefore, the background signal can be determined without separate off-specular measurements. Background contributions in the signal are carefully subtracted before fitting during data reduction.

Prior to NR data collection, samples were carefully aligned on the LIQREF using a goniometer on an adjustable height stage. Sample heights were set to bisect the incident beam after rotating them to be parallel with the source. A separate measurement was taken with the sample removed from the beam’s path to determine the incident intensity through the aluminum can. This value was used to normalize the intensity during data reduction. SNS scientists assisted in creating a template for their reduction software, allowing data to be autoreduced into *Q*_*z*_ versus reflectivity immediately after each run. Tests at each RH were repeated five times to improve statistics and to ensure that the system had reached equilibrium.

## 3 Results and Data Analysis

### 3.1 XPS Analysis

XPS data was processed using CasaXPS.^42^ Fitting C 1s peaks followed recommendations from multiple sources.^43−45^ Due to the electrically insulating silicon supports used in each sample, data was charge-corrected by shifting the C 1s peak to 284.5 eV. A Shirley background was applied to all spectra, and Voigt-type line shapes (i.e., a convolution of Gaussian and Lorentzian) were used for all components.

**Figure 2:**
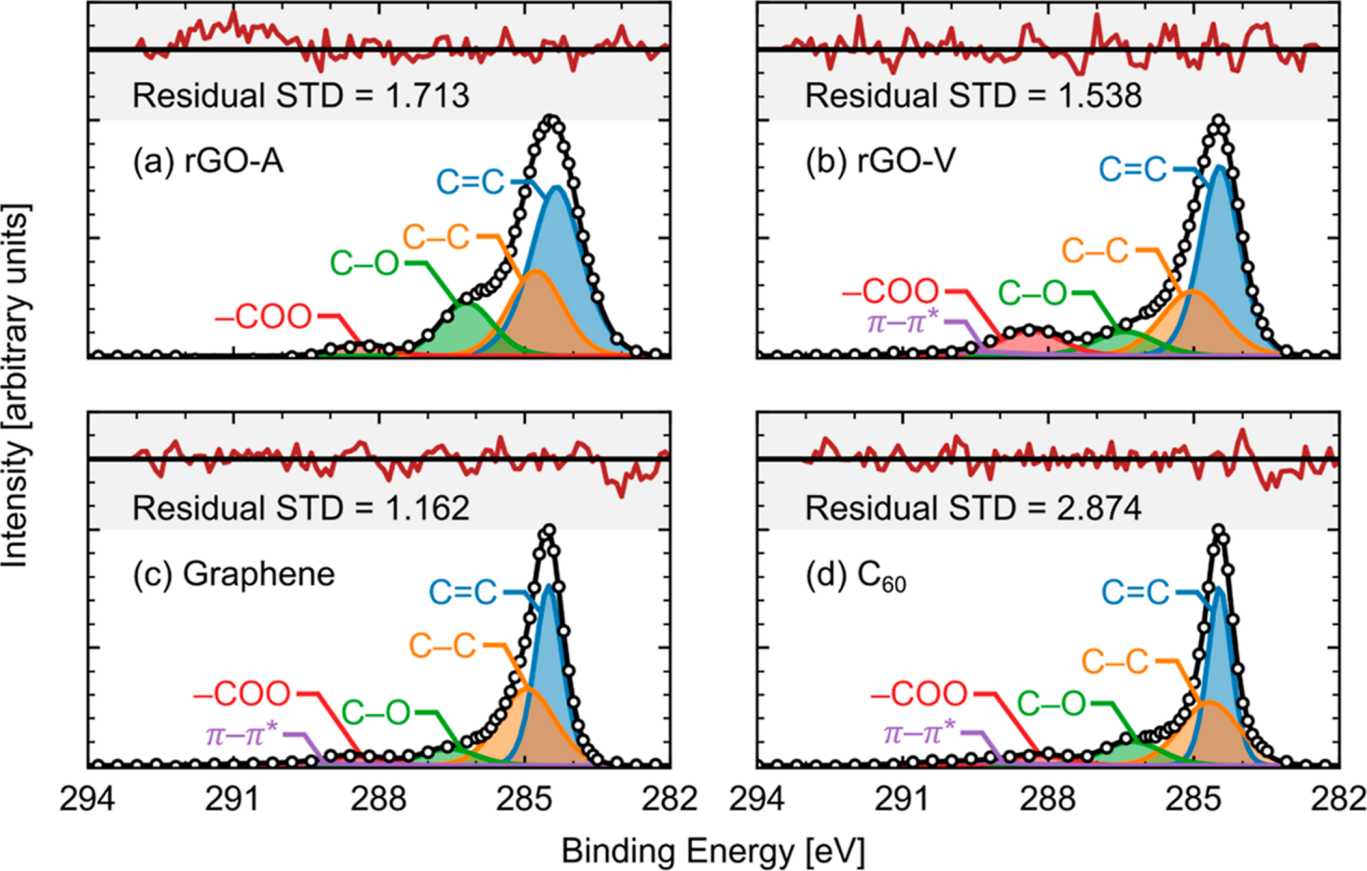
Fitted C 1s peaks for each sample: (a) rGO-A, (b) rGO-V, (c) graphene, and (d) C_60_. The fit envelope is shown as a solid black line beneath the data points, shown as open circles. Note that to improve clarity of the fit envelope outside of the main peak, only one in four data points are shown for binding energies >286 or <284 eV. However, residual values are still calculated using all data points, including those not shown here. Specifics for component peak locations, fwhm, and relative areas are listed in Table 1.

While fitting, constraints were imposed on many of the component peaks. The full width half-maxima (fwhm) of the C–O and –COO groups were constrained to the same value as the C–C peak, which itself was constrained to 0.9–1.6 eV. All peak positions were constrained relative to the C=C peak following ranges taken from the literature.^44,46^ The fitted C 1s spectra are presented in Figure 2, with fitted parameters for each component peak in Table 1. As shown in the figure, the fits are in good agreement with the data.

**Table 1:** XPS Results for Fitted C 1s Component Peaks

group	position (fwhm) [eV]	area [%]
rGO-A		
C=C	284.3 (1.25)	52.64
C–C	284.8 (1.30)	26.71
C–O	286.2 (1.30)	16.83
–COO	288.4 (1.30)	3.82
rGO-V		
C=C	284.5 (0.97)	50.40
C–C	285.0 (1.50)	26.06
C–O	286.4 (1.50)	9.89
–COO	288.4 (1.50)	10.22
π–π*	289.8 (2.89)	3.42
Graphene		
C=C	284.5 (0.68)	45.66
C–C	285.0 (1.35)	38.14
C–O	286.6 (1.35)	8.42
–COO	288.5 (1.35)	4.52
π–π*	289.7 (3.37)	3.25
C_60_		
C=C	284.5 (0.58)	41.45
C–C	284.7 (1.43)	35.22
C–O	286.3 (1.43)	13.48
–COO	288.2 (1.43)	5.67
π–π*	289.8 (2.43)	4.18

### 3.2 Fitting NR Data

**Figure 3:**
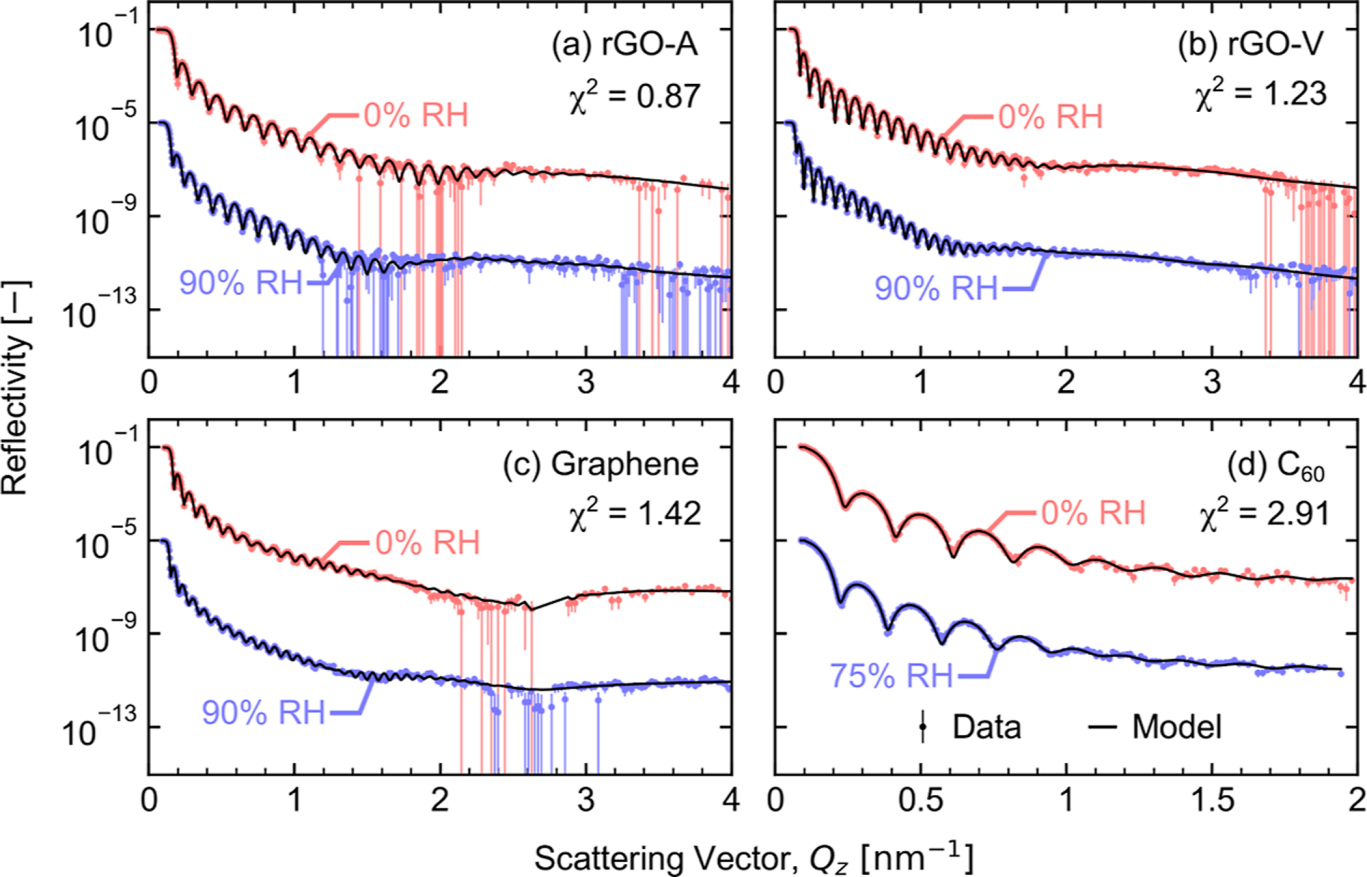
Reflectivity data (symbols) collected at two relative humidities (RHs) for each sample: (a) rGO-A, (b) rGO-V, (c) graphene, and (d) C_60_. Best model fits (black lines) are overlaid for each data set. The χ^2^ statistic provides a measure for the goodness-of-fit, as described in the Supporting Information, Section 3.2. Offsets are used to shift curves in the *y*-direction for improved clarity.

The measured NR data and associated model fits are presented in Figure 3. NR data was fit using Refl1D,^47,48^ a Python package developed at NCNR. As an input, Refl1D takes a model file that constructs an SLD profile by creating a series of adjacent layers, each with their own thickness, real and imaginary SLDs, and interfacial width (i.e., roughness). Known thicknesses, SLDs, and/or roughnesses are specified as constants in the model, while other parameters are fit, holding the value between user-defined limits. Refl1D uses a sample’s SLD model to predict its theoretical reflectivity. Unknown parameters are varied within their given bounds using a DREAM algorithm,^49^ a population-based Markov Chain Monte Carlo method. The DREAM algorithm generates random parameter sets within the supplied bounds. For each parameter set, a normalized χ^2^ is calculated and used to determine the likelihood of fit. A random number is generated, and if it exceeds the likelihood of the parameter set, the set is replaced with one derived from the remaining sets. Thus, after many generations, the distribution of parameter sets represents the probability distribution of the fit. This is used to determine the global best fit, uncertainties in parameters, and correlations between parameters. With a sufficient population size and number of steps, the DREAM algorithm provides consistent predictions for a global optimum^50^ (e.g., the global minimum error between theoretical and experimental reflectivities).

NR experiments for each sample involved both dry and humidified environments, as discussed in Section 2.3. During the fitting process, simultaneous fits were employed to ensure that any layers unaffected by hydration were kept constant between the dry and wet SLD profiles. This included all fitting parameters for the silicon support, native SiO_2_, and bonding layer (when applicable). Constraints were also applied to carbon thicknesses and SLDs. The carbon layers were expected to be mostly rigid and nonporous; however, a slight swelling and decrease in SLD could occur if water absorbed into these layers during high-RH tests. Therefore, the humidified carbon layer thickness and SLD were constrained to be within 10% of their dry values (from 0% RH experiments) in the simultaneous fits. The dry carbon layer SLDs fit to 5.12 × 10^–4^ nm^–2^ for rGO-A, 5.87 × 10^–4^ nm^–2^ for rGO-V, 5.12 × 10^–4^ nm^–2^ for graphene, and 4.76 × 10^–4^ nm^–2^ for C_60_.

The most challenging layer to fit in each sample is the Nafion thin film. All other layers can be reasonably modeled by using a single constant-SLD layer. However, a multilayered model is necessary to properly fit the Nafion if lamellar phase segregation occurs in the polymer. Because it is unclear what Nafion structures to expect, we performed a rigorous study involving up to 120 SLD models per sample to determine Nafion structures near each carbon interface. Models were run in parallel batches using high-performance computing resources at CSM.

The Nafion SLD profiles were modeled using up to three regions:

(i) Interfacial layer(s) closest to the carbon interface. This region was modeled using between 0 and 5 layers, each with a bounded thickness between 0.5 and 2.5 nm, to capture possible lamellae.
(ii) Majority—a single constant SLD layer, between the interfacial layers and a surface layer (when present), with a large thickness range (i.e., 10–100 nm). Hydration in this layer is uniform and isotropic, much like in thick Nafion membranes.
(iii) Surface—a single constant SLD layer at the Nafion–vapor interface. When considered, this layer allows for a hydrophobic “skin” to be captured in the SLD profile. Hydrophobic skins have been observed for Nafion thin films on various substrates but are not currently well understood.^28,51,52^

Each Nafion layer *i* was fit with an independent parameter for its Nafion volume fraction (*V*_Naf,*i*_). The Nafion fraction was used to calculate the SLD of these layers (SLD_*i*_) using a weighted average of the known SLDs for dry Nafion (SLD_Naf_ = 4.16 × 10^–4^ nm^–2^) and liquid water 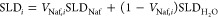
3

Equation 3 assumes that each Nafion layer is exactly composed of two imcompressible phases: Nafion and water. *V*_Naf,*i*_ was limited to a range between 0 and 1.25. When 0 ≤ *V*_Naf,*i*_ ≤ 1, the layer SLD is bound between the SLDs of pure liquid water and dry Nafion (SLD_H_2_O_ ≤ SLD_*i*_ ≤ SLD_Naf_). Allowing *V*_Naf,*i*_ > 1 provides opportunities for profiles to fit any possible regions where Teflon backbones from Nafion molecules may congregate together—resulting in SLD_*i*_ > SLD_Naf_.

Optimal SLD model fits were selected based on statistics and physical relevance to samples. For each simulated SLD profile, a normalized χ^2^ was calculated as
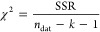
4where *n*_dat_ and *k* are the numbers of data points and fitting parameters used, respectively. SSR is the sum of squared residuals
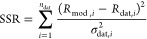
5between the modeled (*R*_mod,*i*_) and measured (*R*_dat,*i*_) reflectivities for each data point *i*. The standard deviation σ_dat,*i*_ is taken from error bars present in the data (Figure 3), which are calculated according to counting statistics. Fits with lower χ^2^ have better agreement with data. Typically, a χ^2^ < 2 signifies a good fit. For LIQREF measurements, where estimated uncertainty values are much lower than on AND/R, χ^2^ < 5 represents a good fit.

From a statistical perspective, SLD model fits with a large number of fitting parameters are likely to produce χ^2^ values lower than those with fewer parameters. To ensure that any reductions in χ^2^ were statistically significant (i.e., more complex fits were not favored because they are a better fit to the random variations in the data, or “noise”), the Bayesian information criterion (BIC) was calculated
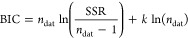
6

As with χ^2^, a lower BIC suggests a better fit. However, in contrast to χ^2^, the BIC penalizes models with a large number of parameters by linearly increasing with *k* based on the statistically expected decrease in χ^2^ with each new parameter. Adding parameters to a model (e.g., more interfacial layers) must sufficiently reduce the SSR to overcome this penalty and be considered a “better” fit. Pixel plots in the Supporting Information demonstrate how BICs changed as the number of interfacial layers was varied, Figures S1–S4.

Fits to NR data are not unique. Two or more qualitatively different SLD profiles may result in similar χ^2^ and/or BIC values. Consequently, all fits having BICs within 7 units of the lowest were evaluated against one another by checking for physical characteristics in each profile. For example, a sample’s wet SLD profile should show a thicker Nafion film when compared to its dry profile because Nafion swells in hydrated environments. Moreover, the amount of Nafion in each sample should be consistent between the wet and the dry SLD profiles. This is verified by calculating the equivalent Nafion thickness
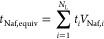
7

The sum in eq 7 is over all interfacial, majority, and surface layers *i* = 1...*N*_L_. The amount of polymer in each layer is the product of the layer’s thickness *t*_*i*_ (nm) and its Nafion volume fraction.

#### 3.2.1 Choice of Best-Fit SLD Profiles

Following the guidelines and physical checks outlined in Section 3.2, the best-fit SLD profiles were determined. Additional detail on how these choices were made for each sample, from their up to 120 modeled SLD profiles, is given in the Supporting Information. Theoretical reflectivities for these profiles show excellent agreement with experimental data (i.e., χ^2^ < 2 for data from AND/R and χ^2^ < 5 for data from LIQREF), as shown in Figure 3. The Supporting Information also includes *t*_Naf,equiv_ histograms in Figure S5, illustrating that the best-fit SLD profiles discussed below are self-consistent between dry and wet environments.

## 4 Discussion

### 4.1 Comparing Carbon Samples to CB

To infer interfacial carbon–Nafion structures in PEMFC CCLs from samples investigated here, characteristics of each carbon are compared to CB. Table 2 shows a summary of samples’ qualitative and quantitative carbon attributes, including roughness, graphiticity, degree of surface oxidation, and hydrophilicity. As demonstrated in the table, a variety of smooth/rough, graphitic/nongraphitic, and hydrophobic/hydrophilic carbons are represented by these samples.

**Table 2:** Summary of Carbon Sample Attributes^a^

sample name	rms roughness [nm] (95% confidence)	C 1s fwhm [eV] (graphiticity)	C:O1:O2, from XPS	hydrophilicity, from the literature	#interfacial layers
This Study					
rGO-A	0.00029 (0.0, 0.3)	1.44 (low)	1:0.21:0.05	high^53,54^	4
rGO-V	0.72 (0.6, 0.8)	1.17 (mid)	1:0.13:0.13	high^53,54^	1
graphene	0.098 (0.0, 0.3)	0.87 (high)	1:0.10:0.05	mid^b^^55,56^	3
C_60_	1.96 (1.1, 2.1)	0.73 (high)	1:0.18:0.07	low^57^	0
CB References					
Vulcan XC-72^d^		1.07 (high)	≈1:0:0	low^c^^58^	
Ketjen EC-600JD^e^		1.46 (low)	1:0.19:0.18	low^c^^58^	

a Roughness, graphiticity, surface chemistry, and hydrophilicity help characterize similarities between samples and the CB (see Section 4.1). Roughness values are taken from the interfacial widths in the best-fit dry SLD profiles.

b Hydrophilicity is thickness dependent and is moderate for the two-sheet graphene film here.

c Hydrophilicity is reported broadly for CBs and is not specific to Vulcan XC-72 or Ketjen EC-600JD.

d XPS information (i.e., C 1s fwhm and C:O1:O2) for Vulcan XC-72 is from Pantea et al.^59^

e XPS information (i.e., C 1s fwhm and C:O1:O2) for Ketjen EC-600JD is from Saito et al.^60^

**Figure 4:**
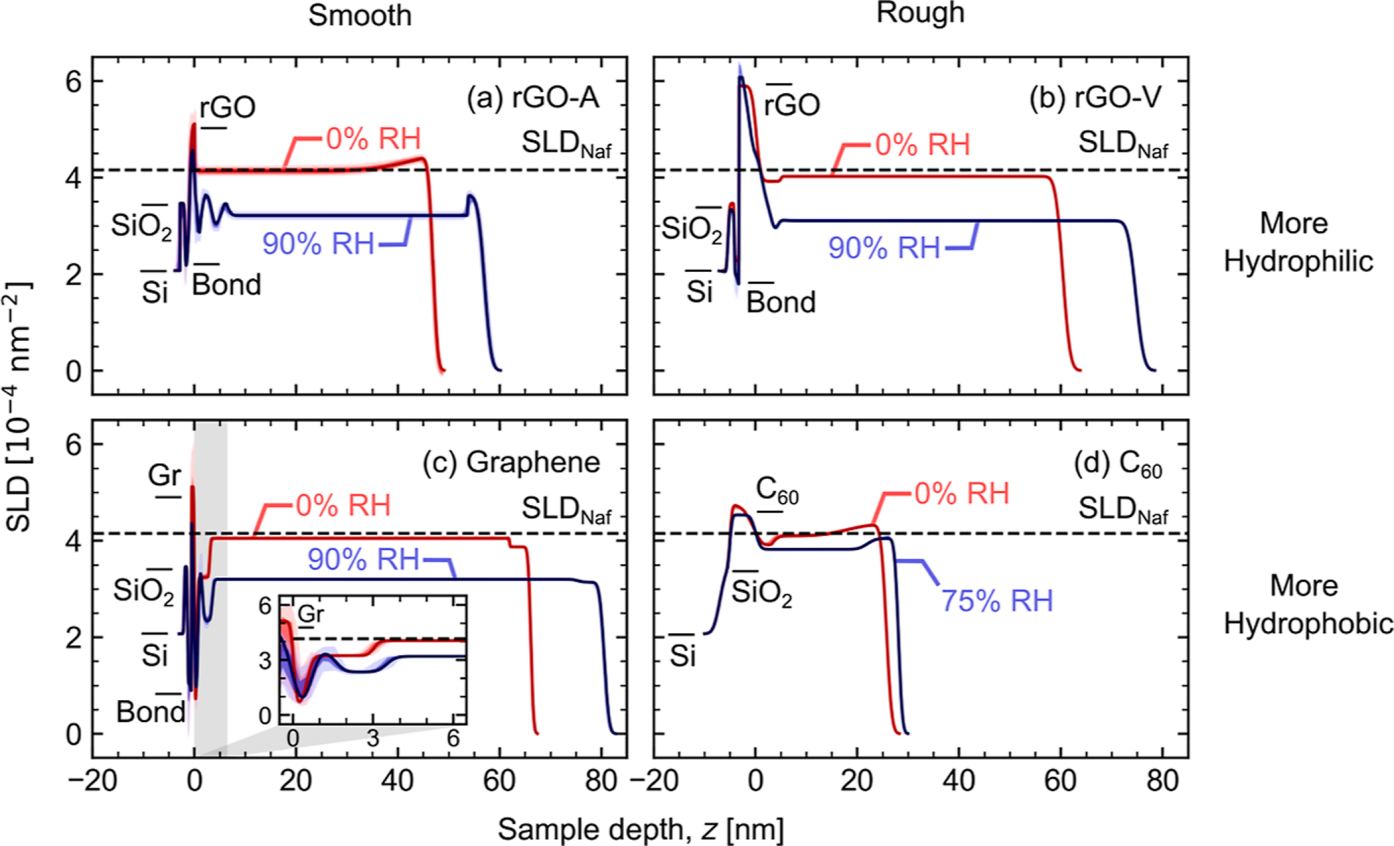
Fitted scattering length density (SLD) profiles for all samples: (a) rGO-A, (b) rGO-V, (c) graphene (Gr), and (d) C_60_—each at two different RH conditions. Best fit profiles are shown as solid lines with shaded regions representing 68 and 95% confidence intervals. Dashed lines show the dry Nafion SLD, i.e., 4.16 × 10^–4^ nm^–2^. SLDs for substrate layers are marked/labeled, including silicon (Si), SiO_2_, bonding layers (Bond), and carbons. The substrate–Nafion interface is defined at *z* = 0 to enable cross-sample comparison.

Common CB supports in PEMFC CCLs are rough due to their granular nature. Large surface areas reduce ionomer poisoning by reducing direct contact between Nafion’s sulfonic acid groups and platinum catalysts.^61,62^ Carbon roughnesses in Table 2 are extracted from the interfacial widths of the best-fit dry SLD profiles, as presented in Figure 4. Results show surface roughness ranged from near zero to 1.96 nm, with the roughest being C_60_ and rGO-V. The 95% confidence intervals (CIs) are also reported for the roughnesses in Table 2, which in some cases are more physically meaningful than the best fit values.

XPS data provide multiple ways to compare carbon samples to CB. The C 1s peak can be used to quantify the carbon’s graphiticity, i.e., how similar it is to graphite, by taking the fwhm of the C 1s peak. A narrow peak suggests a more graphitic carbon due to a larger number of sp^2^ carbons (i.e., C=C bonds) and fewer sp^3^ carbons (C–C bonds).^43^ In addition, qualitative comparisons for surface chemistry can be made using the fitted component peaks. Literature indicates that CB, like graphite, is comprised of sp^2^-hybridized carbon particles.^63^ Pantea et al. present an overview of conductive CBs, showing fwhm’s for C 1s peaks between ≈0.8 and 1.0.^59^ In comparison, Figure 2 provides the fwhm’s for samples’ C 1s peaks in this study. Values in Table 2 demonstrate that C_60_ and graphene are the most graphitic. rGO-A and rGO-V are less graphitic, according to their broadened C 1s peak. XPS data for Vulcan XC-72 and Ketjen EC-600JD in Table 2 are taken from Pantea et al.^59^ and Saito et al.,^60^ respectively. These two CBs are common in PEMFC CCLs, and their range of graphiticity (1.07 to 1.46 eV) is similar to the range covered by our samples (0.73–1.44 eV).

While fitted component peaks provide similarities/differences between carbons, different fitting approaches and inconsistent/incompletely reported details in the literature muddy the accuracy of the comparison. Therefore, only a qualitative comparison is made here, using a ratio of relative component areas—“C:O1:O2” in Table 2. The ratio is normalized by its first value (C), the sum of the C=C and C–C areas. The second and third values are normalized areas from single-oxygen C–O bonds (O1) and two-oxygen –COO bonds (O2), respectively. Using this ratio removes the requirement to refit data in multiple ways (e.g., a combined C=C and C–C peak vs separate) before comparing to the literature. Most CBs have higher carbon purity than the samples here, with undetectable carbon–oxygen bonds (C:O1:O2 ≈ 1:0:0).^59^ Notably, however, Ketjen EC-600JD has demonstrated relatively similar surface chemistries to our samples, C:O1:O2 ≈ 1:0.19:0.18.^60^ This suggests that our samples’ surface chemistries are relevant to PEMFC CCLs, though they are not fully representative of all common CBs used in PEMFCs.

Hydrophilicity can depend on multiple factors, such as texture and chemical makeup. From the previous literature, the carbon materials in this study provide a range of hydrophobic and hydrophilic properties. For example, rGO and C_60_ are generally hydrophilic and hydrophobic, respectively.^53,54,57^ Graphene has demonstrated a thickness-dependent hydrophilicity, with films less than three sheets thick resulting in more hydrophilic surfaces.^55,56^ In comparison, CB supports are predominately hydrophobic.^58^

Considering all characteristics presented in Table 2, C_60_ shares the most similarities with CCL CBs. The C_60_ sample is rough, graphitic, and hydrophobic. In contrast, rGO-A is the least similar to CB due to its lower graphiticity and smooth hydrophilic surface. Remaining samples are between these two extremes. rGO-V is relatively rough but has a less graphitic, more hydrophilic surface. On the other hand, graphene is more graphitic but is smooth with a moderate hydrophilicity. Consequently, Nafion on C_60_ can arguably be interpreted as most similar to Nafion on CB supports, while the rGO-V and graphene can be used to determine which characteristic is more relevant.

### 4.2 Substrate-Specific Nafion Interface Structures

The best-fit SLD profiles for each sample are shown in Figure 4. Results illustrate that Nafion thin films took on a variety of structures near carbon interfaces. For the smooth samples, rGO-A and graphene [panels (a) and (c), respectively], the hydrated profiles clearly show lamellar phase segregation near the substrate. In contrast, the rough rGO-V and C_60_ samples [panels (b) and (d), respectively] show little to no layered structure in the humidified films. Including a surface layer at the Nafion–air interface in our fitting routine benefited some, but not all, of the fits, as shown by the SLD profiles in Figure 4. Similar surface layers are present in previous literature.^28,51,52^ The lack of any apparent pattern when these layers are observed via NR introduces the possibility that these layers are an artifact of systematic errors in the data or fitting. While a concrete explanation for hydrophobic “skins” at the Nafion–air interface remains an open research question, the current study is centered on understanding the carbon–Nafion interface, which is assumed to be unaffected by these surface layers.

Table 2 includes a column for the number of interfacial Nafion layers observed on each carbon. Smoother carbons with at least moderate hydrophilicity in the table correlate with a greater number of phase-segregated interfacial lamellae than hydrophobic carbons. Here, we assume that graphene is moderately hydrophilic because, according to its SLD profiles, it is roughly two sheets thick.^55,56^ Previous literature has also concluded that lamellar phase segregation in thin-film Nafion is caused by hydrophilic surfaces.^29,31,34^ However, Nafion on rGO-V (Figure 4b) deviates from the other hydrophilic samples and has only one thin interfacial layer with marginally more water than its majority layer. This is likely due to differences in surface roughness.

There are two possible explanations for the lack of observed lamellae in rGO-V. As shown in Table 2, rGO-V is at least twice as rough as the rGO-A and graphene samples. One theory is that rough surfaces may inhibit the ordered lamellar phase segregation, in favor of a more isotropic phase segregation similar to that of bulk or majority layer Nafion. Alternatively, it is possible that layered structures follow the rough rGO-V surface topology, forming over relatively large lateral distances. In this case, the layered interface would not be observable with NR because measurements average over large (hundreds of μm) lateral distances in the nominal plane of the sample.^64^

Some details in Figure 4b suggest that a layered interface is present in rGO-V but is not fully resolved in the SLD profile. To start, the interfacial width increases in the 90% RH profile compared to the 0% RH profile, which could be explained by swelling lamellae when Nafion is hydrated. The inset of Figure 4c illustrates what swelling lamellae look like when the layers are more resolved. In the 0% RH graphene profile, the interfacial lamellar region extends roughly 3 nm from the substrate surface, while it is closer to 4 nm in the 90% RH profile. In further support of a lamellar interface in rGO-V, differences between the dry and wet profiles could be interpreted as three lamellae. At *z* < 0, the wet profile dips below the dry profile, indicating higher water uptake. Then, the profiles are equal, indicating no water uptake, before the 90% RH profile dips again for another water-rich layer. Last, the hydrated rGO-V profile shows a thin, slightly water-rich layer at *z* ≈ 5 nm. This could be the last phase-segregated layer of a multilayered interface for this sample with the other lamellae, closer to the interface, averaged out by surface roughness. The distance away from the surface roughly aligns with the last water-rich interfacial layer of rGO-A [panel (a)]. However, the hydrated rGO-A profile also shows another water-poor layer at *z* ≈ 7 nm. Consequently, if rGO-V does have a “hidden” layered interface, it likely has a thinner bilayer period or has fewer layers compared to observations near rGO-A.

Results for C_60_, on the other hand, support hydrophobic and rough carbon surfaces limiting lamellar phase segregation in hydrated Nafion thin films. Compared to rGO-V, there is stronger evidence to reject the possibility of a “hidden” multilayered interface in the C_60_ sample. First, the interfacial width between C_60_ and Nafion is consistent between the dry and wet profiles and does not swell. Additionally, similarly rough C_60_ surfaces have been mapped using scanning tunneling microscopy that show variations in surface topology occur on lateral length scales less than 1 nm.^65^ Nafion is a large molecule and would likely not be able to locally orient and follow rough surfaces over such short lateral distances. The single interfacial layer in the dry C_60_ profile can also be explained without considering phase segregated Nafion. The low-SLD interface is likely either porous Nafion or residual water since the sample was only dried using desiccant and without heating. At 75% RH, either the majority layer hydrates to the same level as the residual water or the porosity is filled in with expanding hydrated Nafion.

With C_60_ being most similar to CB, we infer that thin-film Nafion in PEMFCs does not exhibit lamellar phase segregation. This motivates the question: if multilayered nanostructures are not present in CCLs, what causes reduced species mobilities and increased transport resistances? These can still be attributed to lower water uptake and confinement effects compared to bulk Nafion. Thin-film Nafion on silicon substrates can be split into two regions: an interfacial region with lamellar phase segregation and an outer majority layer with isotropic phase segregation. If lamellar structures were the only determining factor in species transport limitations, then transport in the majority layer would be similar to transport in bulk membranes. However, DeCaluwe et al. demonstrated that reduced mobility is still present in homogeneously hydrated majority layers.^28^ Furthermore, modeling work from Randall and DeCaluwe displayed good agreement to data from low-Pt-loaded PEMFCs when the models incorporated ionomer structure–property relationships assuming uniformly hydrated CCL Nafion at carbon interfaces.^66,67^

### 4.3 Nafion at Carbon vs Silicon Interfaces

Although results in Figure 4 and Table 2 suggest that lamellar structures are not prevalent in CCL Nafion, much can still be learned from samples with multilayered interfacial structures. Literature contains a multitude of insightful articles for multilayered Nafion on silicon substrates.^17,28−30^ Comparing our results to these previous data can inform how to use silicon–Nafion experiments to guide future PEMFC research. In this section, we compare structure, water uptake, and species mobility between our carbon–Nafion samples and silicon–Nafion samples from ref (28).

#### 4.3.1 Impacts on Phase Segregation

**Figure 5:**
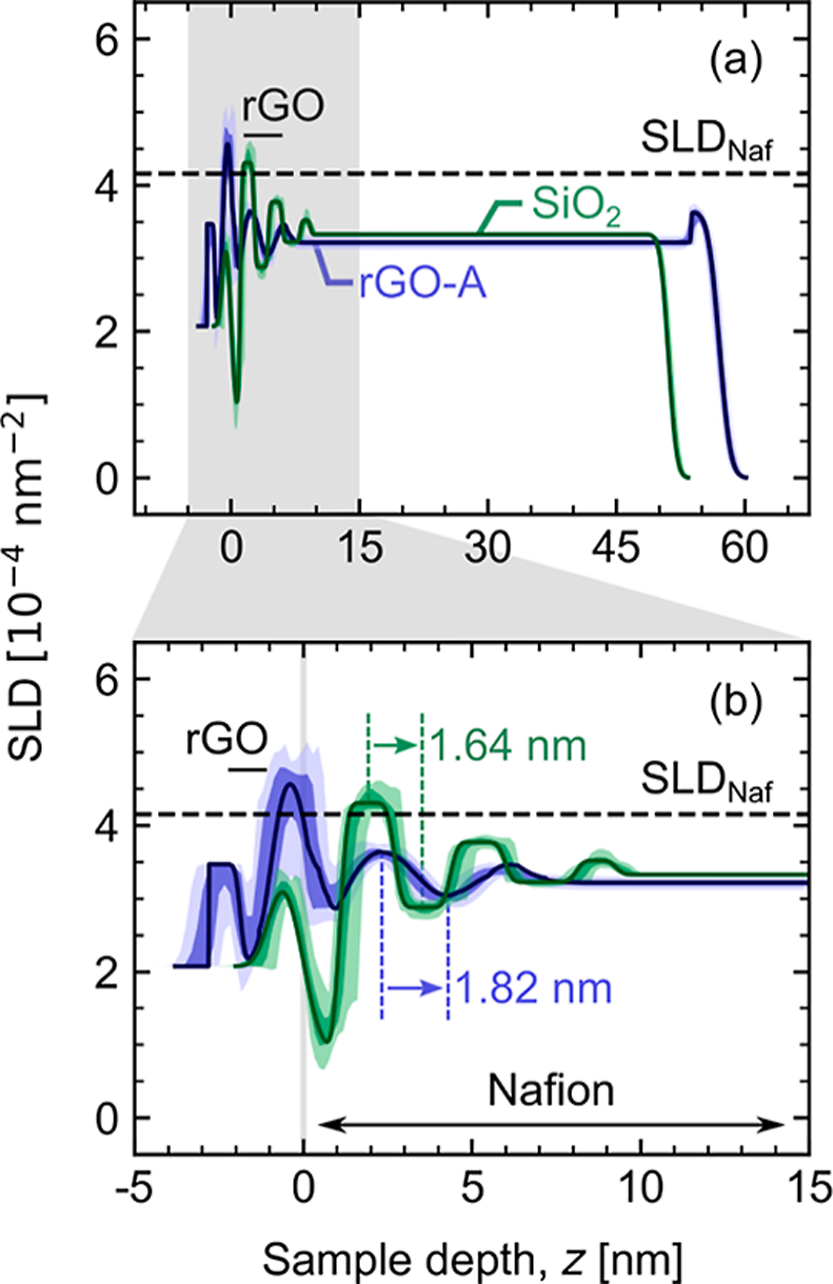
Overlaid SLD profiles for hydrated Nafion on rGO-A (this study) and SiO_2_ (ref (28)) substrates. Results demonstrate that even when multilayered phase segregation occurs near carbon interfaces, it is less persistent compared to Nafion on silicon. Hydration in the majority layers, however, is approximately equal for similarly thick films.

Hydrated Nafion on rGO-A showed the most resemblance to Nafion on silicon substrates. Figure 5 overlays 90% RH SLD profiles for similarly thick Nafion films on rGO-A (this study) and SiO_2_ (from ref (28)). In panel (a), SLDs for the majority Nafion layers are nearly indistinguishable, suggesting similar water uptake in both. Despite this similarity, the cropped interfacial region [panel (b)] demonstrates structural differences. Despite similar surface roughness of the underlying substrate, more extreme SLD oscillations indicate a stronger lamellar phase segregation for Nafion on SiO_2_. This is consistent with fewer lamellae in the rGO-A profile.

Table 3 presents the thicknesses and Nafion volume fractions for the Nafion layers in Figure 5. These values further emphasize a weaker lamellar phase segregation for Nafion on rGO-A. The interfacial region near rGO-A persists only ≈6.5 nm beyond the substrate surface. On SiO_2_, this region is ≈9.3 nm—a 43% increase. Moreover, Nafion fractions for rGO-A are all within ±0.08 of the majority layer Nafion fraction. For SiO_2_, this interval is much higher, ±0.52, highlighting how different the water-rich and water-poor layers are between the two samples. Last, the interfacial lamellae are not observed on dehydrated (0% RH) rGO-A, whereas residual layered structures (roughly three layers) are observed in dehydrated Nafion on SiO_2_. A reasonable explanation for this, based on previously discussed trends, is that SiO_2_ is more hydrophilic than rGO-A. Silicon substrates with native oxide layers show water contact angles as low as 20°.^68^ For rGO, contact angles are closer to ≈50–75°.^53^ The hydrophilicity of SiO_2_ may lead to stronger bonding between the sulfonic acid functional groups in the Nafion and the SiO_2_ substrate compared to weaker, impersistent bonding at the less hydrophilic rGO-A interface.

**Table 3:** Layer Thicknesses and Nafion Fractions from the Hydrated rGO-A and SiO_2_ SLD Profiles^a^

layer number	thickness [nm]		Nafion fraction [—]	
	rGO-A	SiO_2_	rGO-A	SiO_2_
1	1.21	1.09	0.75	0.30
2	1.86	1.62	0.88	1.03
3	1.78	1.66	0.76	0.73
4	1.61	1.62	0.84	0.91
5		2.11		0.80
6		1.18		0.86
majority	45.17	41.79	0.80	0.82
surface	5.34		0.86	

a Values for SiO_2_ are from ref (28).

#### 4.3.2 Influence on Water Uptake

Local Nafion hydration can be discussed in terms of either water volume fractions (*V*_H_2_O,*i*_) or water uptake . Here,  is calculated from the high-RH SLD profiles’ fitted *V*_Naf,*i*_
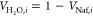
8

A layer’s water uptake is a function of its water volume fraction
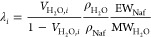
9

In eq 9,  and ρ_Naf_ are the densities of liquid water and dry Nafion, respectively.  is the molecular weight of H_2_O, and EW_Naf_ is the Nafion equivalent weight, i.e., the mass of dry Nafion per mole of sulfonic acid groups.  in this study.

**Figure 6:**
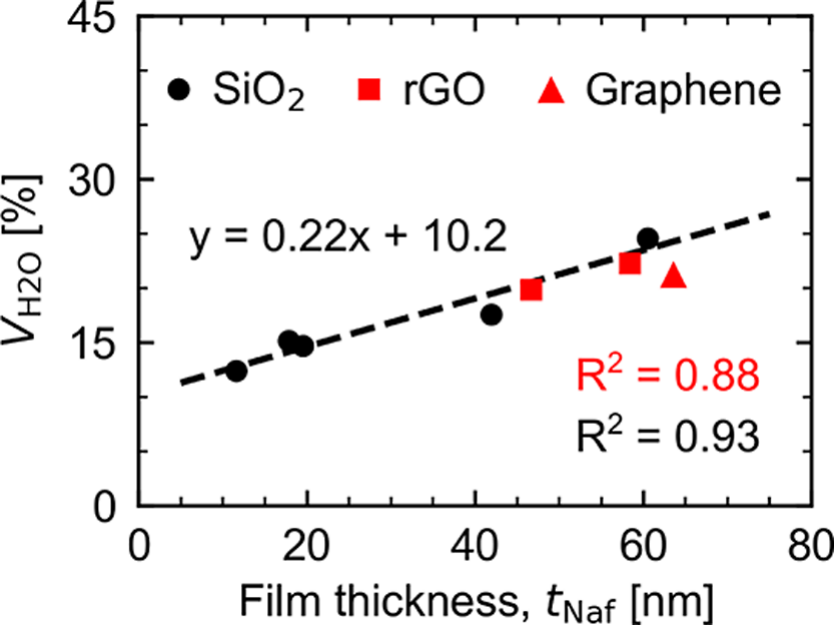
Majority layer Nafion water volume fractions as a function of the film thickness. All data is from experiments at 90% RH. SiO_2_ data (black circles) is from ref (28). Data for Nafion on carbon substrates (this study) are shown with red symbols. The trendline is fit using only the SiO_2_ data but still shows good agreement with the added carbon data. Error bars are plotted but are not visible because they are within the size of the symbols.

Majority layer water volume fractions from silicon–Nafion experiments in the thin-film regime (i.e., 10 nm ≤ *t*_Naf,equiv_ ≤ 60 nm) have a linear dependence on film thickness at 90% RH.^28^Figure 6 illustrates how well this same trend fits for carbon–Nafion samples at the same RH. In the figure, the trendline is fit exclusively using the majority layers from SiO_2_ data (from ref (28)), resulting in *R*^2^ = 0.93. Without changing the slope or intercept of this trendline, adding data for the majority layers on rGO-A, rGO-V, and graphene only causes a small reduction to the fit quality, *R*^2^ = 0.88. It is worth emphasizing that Figure 6 only represents  trends for the majority Nafion layers, rather than the average water uptake, which would include interfacial and/or surface layers. This suggests that while structures at silicon–Nafion and carbon–Nafion interfaces are significantly different, water uptake in the majority Nafion layer is mostly independent of the support material. Consequently, in the thin-film regime, the majority Nafion layer appears unaffected by any layered interfacial nanostructures resulting from substrate interactions.

The C_60_ sample was left out of Figure 6 because it was hydrated in a 75% RH environment, rather than 90% RH. To compare how water uptake in this sample fits with trends from SiO_2_, a correlation has to be made to account for differences in RH. It is well known that λ varies nonlinearly with water activity in bulk Nafion films. Springer et al. fit a cubic polynomial to data from bulk Nafion membranes in 1991^69^
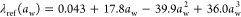
10

**Figure 7:**
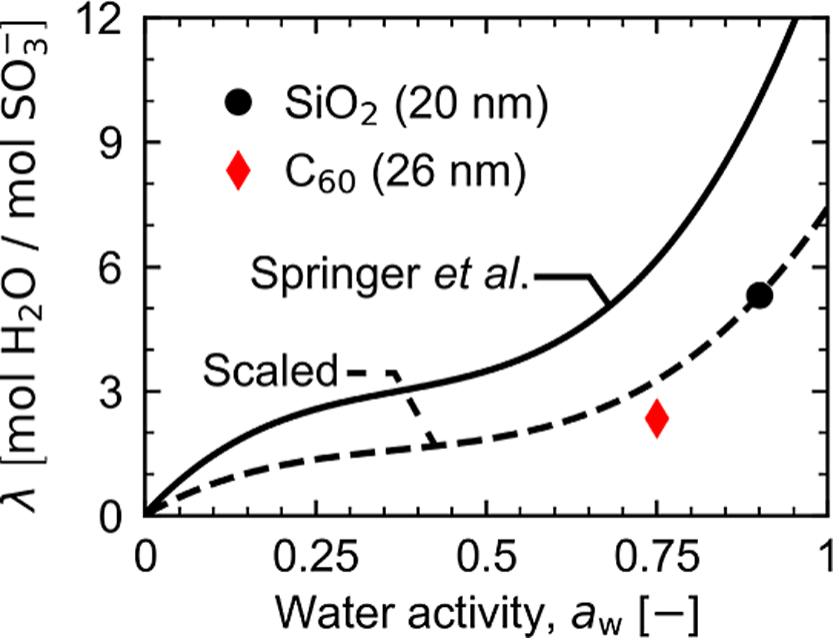
Majority layer water uptake versus water activity for Nafion thin films on silicon (black circle) and C_60_ (red diamond). The Nafion isotherm (eq 10, solid black line) from Springer et al.^69^ is shown for comparison against bulk membranes. The “scaled” isotherm (dashed line) scales the bulk Nafion isotherm by a constant multiplier (λ_maj,SiO_2__/λ_ref_(0.9) ≈ 0.53). Error bars are plotted but are not visible because they are within the size of the symbols.

The curve drawn out by eq 10 is generally regarded as the most common isotherm for thick Nafion membranes.^70^Figure 7 shows this isotherm as a solid black line. Thin-film Nafion has generally followed this form for different substrates and thicknesses, but typically with λ lower than that observed in thick membranes at the same *a*_w_.^71,72^ We therefore estimate λ as a function of *a*_w_ for the C_60_ sample by scaling the Springer polynomial to coincide with λ from a sample on SiO_2_ with a similarly thick majority Nafion layer (20 nm). The resulting isotherm is shown as a dashed black line. Data for the majority layer on C_60_ is only slightly below this scaled isotherm, indicating that the water uptake in the Nafion majority layer for the C_60_ sample is similar to what would be expected for a similarly thick Nafion film on SiO_2_ at this *a*_w_, according to the assumed scaling. This highlights the importance of water management in PEMFC CCLs; PEMFC design and operation must simultaneously maintain a high local RH in the CCL while also limiting flooding.

## 5 Conclusions

Species transport in CCL Nafion is a significant source of losses in low-Pt-loaded PEMFCs, but a thorough mechanistic understanding for these losses is lacking in the literature. Layered nanostructures at silicon–Nafion interfaces, from NR experiments, have been correlated with reduced ionic conductivities in thin-film Nafion.^28^ Nevertheless, Nafion in PEMFC CCLs bonds to CB and cannot be assumed to be the same as Nafion on silicon supports. In this study, we therefore applied in situ NR experiments to further investigate structures at carbon–Nafion interfaces, including reduced graphene oxide (rGO), graphene, and C_60_.

In agreement with Dura et al.,^29^ Kim et al.,^31^ and Ito et al.,^34^ hydrophilic surfaces were correlated with lamellar phase segregation in interfacial Nafion. However, this was more true for smooth surfaces than for rough ones. In the case of rGO-V, a hydrophilic and rough sample, multilayered interfacial phase segregation was not observed in its SLD profiles. This could suggest one of two conclusions: (i) rough interfaces disrupt lamellar phase segregation or (ii) layered interfaces cannot be resolved on rough surfaces because NR measurements average over them. The latter conclusion is more probable for rGO-V due to observed differences between its dry and wet SLD profiles. However, the former may be more likely for C_60_ based on surface topology measurements in the literature showing C_60_ roughnesses that do not span large lateral distances.^65^

Each carbon sample was compared to CB using XPS and NR to extract surface characteristics. C_60_ demonstrated a strong likeness to CB due to its rough, graphitic, and hydrophobic surface. Because hydrated Nafion on C_60_ indicated no lamellar phase segregation, it is likely that CCL Nafion also has little to no layered structuring. In further support, although rGO-A displayed the strongest multilayered phase segregation,  from its water-rich and water-poor layers only deviated by ±0.08 from its majority layer. Therefore, even if interfacial lamellae were present in CCL Nafion, they would likely have less influence on water uptake and species transport compared with structures observed on silicon supports.

Because silicon–Nafion NR experiments are more abundant in the literature than carbon–Nafion experiments, we conducted a comparison here. Results demonstrate that the relationship between water uptake and film thickness in the majority Nafion layers on carbon matches that on silicon and is not impacted by Nafion’s interfacial structure. This implies that data from SiO_2_ studies may still provide useful insight into CCL Nafion. Some of the present authors have previously presented similar hypotheses, using water uptake and reduced species mobilities inferred exclusively from Nafion on SiO_2_ (from ref (28)) in physics-based models to predict PEMFC performance as a function of Pt loading.^66,67^ Outcomes showed excellent agreement with low-Pt-loaded PEMFC polarization data, indicating the suitability of majority layer Nafion on silicon supports as a reasonable surrogate for that in the PEMFC CCL.

## Supporting Information Available

Pixel plots of rGO-A model fits, rGO-V model fits, C_60_ model fits, and graphene model fits and histograms of equivalent Nafion thicknesses (PDF)

## Footnotes

a Certain commercial equipment, instruments, or materials (or suppliers, or software, etc.) are identified in this paper to foster understanding. Such identification does not imply recommendation or endorsement by the National Institute of Standards and Technology, nor does it imply that the materials or equipment identified are necessarily the best available for the purpose.
