# Supplementary material for: Morphology of Thin-Film Nafion on Carbon as an Analogue of Fuel Cell Catalyst Layers
Source: ACS Appl Mater Interfaces. 2024 Jan 11;16(3):3311–24. doi: 10.1021/acsami.3c14912 (PMC10811627; doi:10.1021/acsami.3c14912)
Supplement: Supplementary file 1 — am3c14912_si_001.pdf [file am3c14912_si_001.pdf]

Supplementary Information: Morphology of thin film Nafion on carbon as analogs of fuel cell catalyst layers

Corey R. Randall[a], Lianfeng Zou[c], Howard Wang[d], Jingshu Hui[e], Joaquín Rodríguez-López[e], Melodie Chen-Glasser[a], Joseph A. Dura[b,*], Steven C. DeCaluwe[a,**]

[a]*Colorado School of Mines: Golden, Colorado, 80401, United States*
[b]*NIST Center for Neutron Research: Gaithersburg, Maryland, 20899, United States*
[c]*Clean Nano Energy Center, State Key Laboratory of Metastable Materials Science and Technology, Yanshan University: Qinhuangdao, Hebei, 066004, China*
[d]*University of Maryland: College Park, Maryland, 20742, United States*
[e]*University of Illinois at Urbana-Champaign: Urbana, Illinois, 61801, United States*
[f]*National Renewable Energy Laboratory: Golden, Colorado, 80401, United States*

1. Neutron Reflectometry Data Fitting

Fitting and interpreting neutron reflectometry (NR) data is challenging because models are not unique. Two different scattering length density (SLD) profiles can provide similar reflectivity curves, resulting in equally good statistical agreement to the data. Therefore, in fitting our NR data, we applied a rigorous and robust approach to model and compare SLD profiles. The process involved simultaneously fitting data taken in both dry and humidified environments, and running a large number of models with varying complexity. Simultaneous fits ensure models are self consistent, i.e., the thicknesses, SLDs, and roughnesses for layers that are not expected to change between the dry and wet environments are kept constant.

In total, we ran up to 120 SLD model fits per sample. Fits covered a range of possible layers/microstructures that could be present. For example, we considered a bonding layer which may or may not exist between the silicon supports and carbon substrates. Furthermore, we ran fits to consider whether or not a surface layer was present at the vapor interface. The combination for cases with or without a bonding layer, and with or without a surface layer, produces four distinct parametric studies for each sample. In each parametric study, the number of interfacial layers for the dry and wet SLD profiles were independent, and the Nafion layers between the two profiles were not constrained to one another. Additionally, all interfacial layers were fit with the same SLD ranges. Therefore, adjacent layers could fit to the same SLD, thereby representing a model with fewer layers.

Fits with more layers and more complexity have a greater likelihood of producing results with a good statistical agreement to data than fits with fewer layers and less complexity. This is because introducing more layers to a fit results in more fitting parameters, which can start to fit random variations and noise in the data. For each model, we calculated a normalized χ^2 and the Bayesian Information Criterion (BIC). The mathematic definitions for the χ^2 and BIC statistics are given in equations (4) and (6). So long as SLD models stay physically representative of a sample, the model with the lowest BIC generally represents the best to the data. The following subsections discuss the BIC pixel plots for all models and all samples in this study.

*Corresponding Author, dura@nist.gov
**Corresponding Author, decaluwe@mines.edu

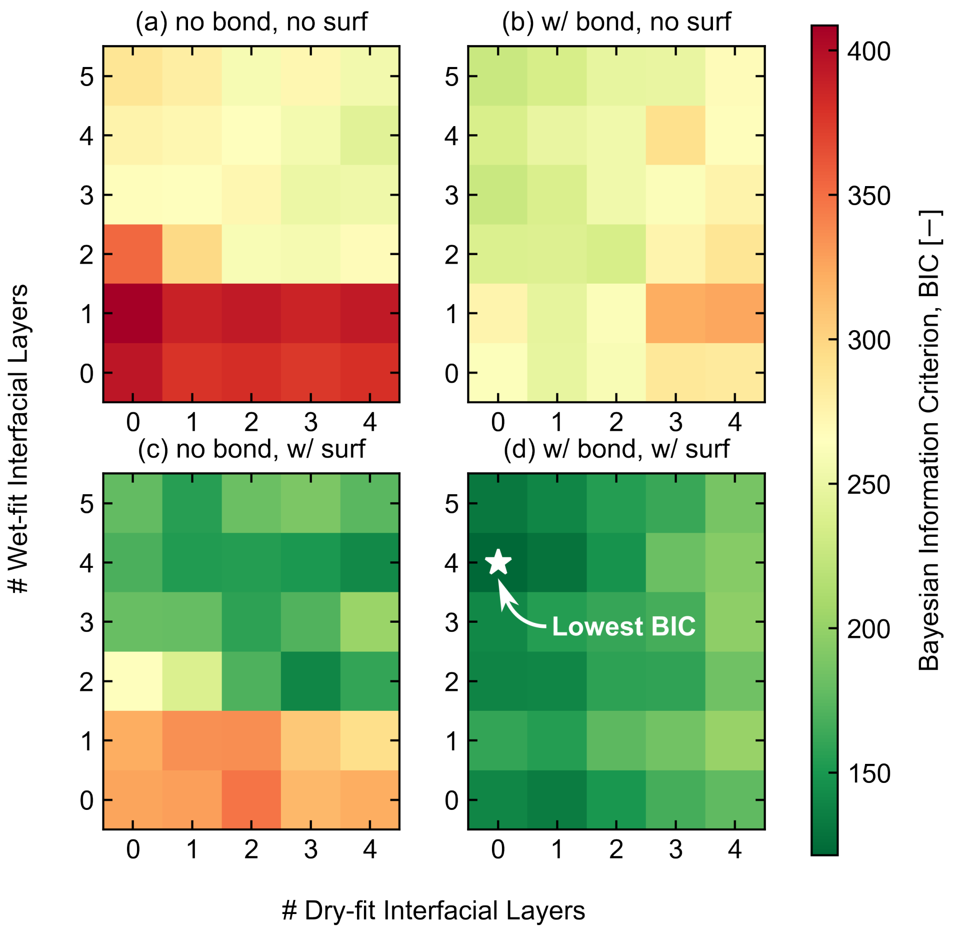

Figure S1: Pixel plots for rGO-A model fits with and without a bonding and/or surface layer. Each pixel represents the BIC statistic for a best fit SLD profile with the specified number of interfacial layers. The number of interfacial layers between the wet and dry data sets are independent of one another. The lowest BIC, i.e. the best fit, is marked with a star.

1.1. rGO-A Fitting Summary

Figure S1 shows pixel plots for BICs calculated from 120 different SLD model fits for the rGO-A sample. The number of interfacial layers were varied between 0–5 for the wet profile and between 0–4 for the dry profile. As shown in the figure, the lowest BICs were produced for models that included both a bonding and a surface layer, panel (d). The lowest BIC is marked in Figure S1(d) with a white star, indicating that the best model fit had zero interfacial layers in the dry profile, and four in the wet profile. The SLD profiles from this fit are shown in Figure 4(a). As shown in the profiles, all fit layers: bonding, surface, and all four wet interfacial layers, are distinct.

1.2. rGO-V Fitting Summary

Figure S2 shows model fits for rGO-V had the lowest BICs when a bonding layer was present. Similar BICs are observed in Figures S2(b) and (d) because adding a surface layer had a negligible difference. In contrast, Figures S1(b) and (d) show significant differences, emphasizing the importance of the surface layer in the rGO-A models.

The lowest BIC for rGO-V is marked with a white star in Figure S2(b). This suggests a model with one dry and three wet interfacial layers produces the best fit. The profiles from this fit are shown in Figure 4(b). Compared to the rGO-A sample, not all layers are distinctly visible in the SLD profile. Instead, only one interfacial layer is distinct in

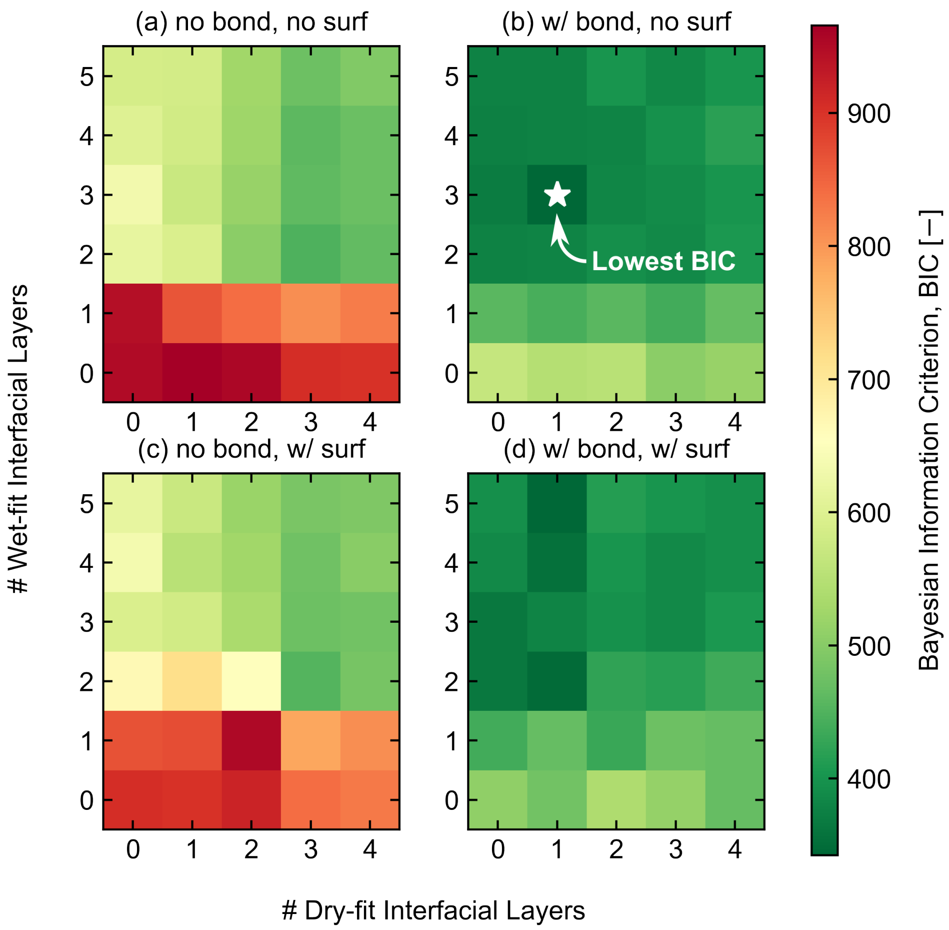

Figure S2: Pixel plots for rGO-V model fits with and without a bonding and/or surface layer. Each pixel represents the BIC statistic for a best fit SLD profile with the specified number of interfacial layers. The number of interfacial layers between the wet and dry data sets are independent of one another. The lowest BIC, i.e. the best fit, is marked with a star.

each profile. The model uses the remaining two wet "interfacial layers" to approximate a relatively rough surface. It is worth noting that some BICs in Figure S2(d) were within seven units of the lowest. Although these fits included a surface layer, it was not distinct in the profile. Rather, it fit at nearly the same value as the majority layer. In addition, the interfacial Nafion region for these "alternate" best fits showed the same general trend as the fit from the lowest BIC.

1.3. C_{60} Fitting Summary

The C_{60} sample required fewer models to find the lowest BIC because the profile that the model needed to capture was less complex, as shown in Figure 4. Therefore, rather than needing to fit 120 models, the lowest BIC was identified using 64 models. Figure S3 shows that C_{60} required a surface layer, but had no significant benefit from adding a bonding layer. The best fit is marked in Figure S3(c) with one dry and zero wet interfacial layers. As with the other samples, any BICs within seven units of the lowest were also investigated. However, they demonstrated no notable differences compared to the fit marked by the white star.

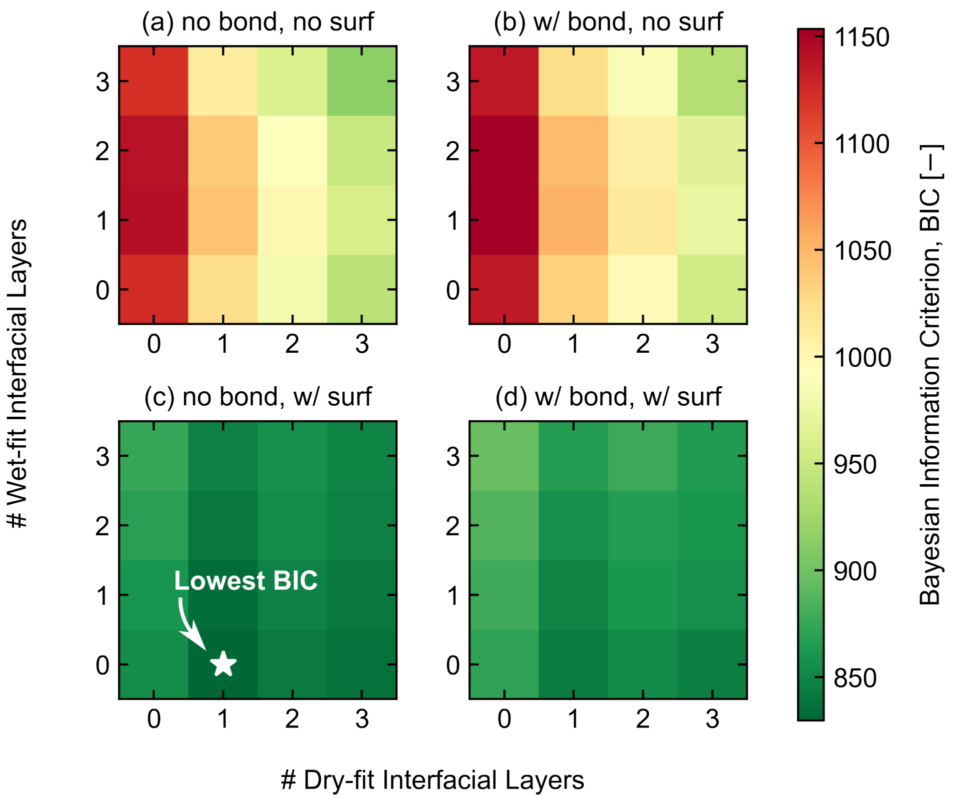

Figure S3: Pixel plots for C_{60} model fits with and without a bonding and/or surface layer. Each pixel represents the BIC statistic for a best fit SLD profile with the specified number of interfacial layers. The number of interfacial layers between the wet and dry data sets are independent of one another. The lowest BIC, i.e. the best fit, is marked with a star.

1.4. Graphene Fitting Summary

The graphene sample was the last to be fit. After learning from fitting the other samples, it was clear that the SLDs for the bonding and surface layers would not be distinct in the overall profile if they did not significantly benefit the fit. Therefore, rather than performing four parametric studies that included all combinations of with and without bonding/surface layers, only the most complex case was run (i.e., with both bonding and surface layers). A pixel plot of the BICs is given in Figure S4. The lowest BIC is marked, indicating the best fit required two dry and three wet interfacial layers. These are clearly shown in the profiles in Figure 4. The surface layers in these profiles are less distinguishable from the majority layer than in other fits. Especially in the case of the wet profile, the majority and surface layer SLDs show essentially no difference. Although it is possible that removing the surface layer from this fit could have produced a marginal decrease in the BIC, we make no strong arguments about surface layers in this sample. Rather, we assume that it is spurious compared to surface layers present in the other samples' SLD profiles.

2. Statistics on Thicknesses and Water Volume Fractions

As explained above, theoretical reflectivities calculated from modeled SLD profiles are not unique. Therefore, a fit SLD profile may be non-physical but still have good statistical agreement with data. Consequently, all fits were scrutinized for their physicality by examining Nafion conservation between dry and wet profiles. While fitting, Nafion

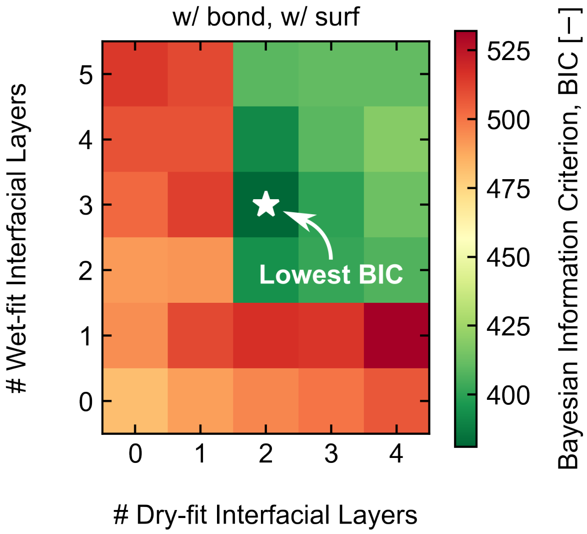

Figure S4: Pixel plots for graphene model fits with and without a bonding and/or surface layer. Each pixel represents the BIC statistic for a best fit SLD profile with the specified number of interfacial layers. The number of interfacial layers between the wet and dry data sets are independent of one another. The lowest BIC, i.e. the best fit, is marked with a star.

regions between dry and wet fits were not constrained. Instead, fitted profiles were post processed to check for Nafion conservation.

An equivalent thickness can be calculated using the Nafion volume fraction and thickness of each layer, see equation (7). Although the single best-fit dry and wet SLD profiles can be checked against one another for Nafion conservation, it is more informative to get statistics on this calculation. To do this, the top 50,000 fits for each sample (i.e., stored Refl1D fits near the lowest χ^2) were used to obtain a distribution of equivalent thicknesses t_{Naf}. The results are shown with histograms in Figure S5. For each sample, the dry and wet equivalent thicknesses were all within each others 68% confidence intervals. The same 50,000 fits per sample can also be used to get statistics on other calculated values. For example, tables in Figure S5 show confidence intervals (CIs) for the hydrated samples' average water volume fractions. The small intervals demonstrate why error bars were not visible when plotted with $V_{\mathrm{H_2O},i}$ and λ_i data in the main manuscript.

An additional analysis was performed for the graphene sample concerning the interfacial Nafion. The dry profile showed only two interfacial layers while the wet showed three. Between the two SLD profiles, it appeared that the interfacial layer closest to the graphene did not significantly change. This suggests that interfacial water is likely trapped in the film, even when in equilibrium with a 0% RH environment. The second interfacial layer (L2) however, is relatively thick in the dry profile – compared to other samples. We hypothesized that the structure in this L2 layer is independent of what occurs in the majority layer. To test this, we performed an equivalent Nafion thickness calculation using the second and third interfacial layers of the wet profile (L2 and L3), and compared it to the L2 equivalent thickness of the dry profile. The results are shown with the remaining graphene calculations in Figure S5. The best-fit values and confidence intervals show good agreement, suggesting that the confined interfacial Nafion is independent from the rest of the film.

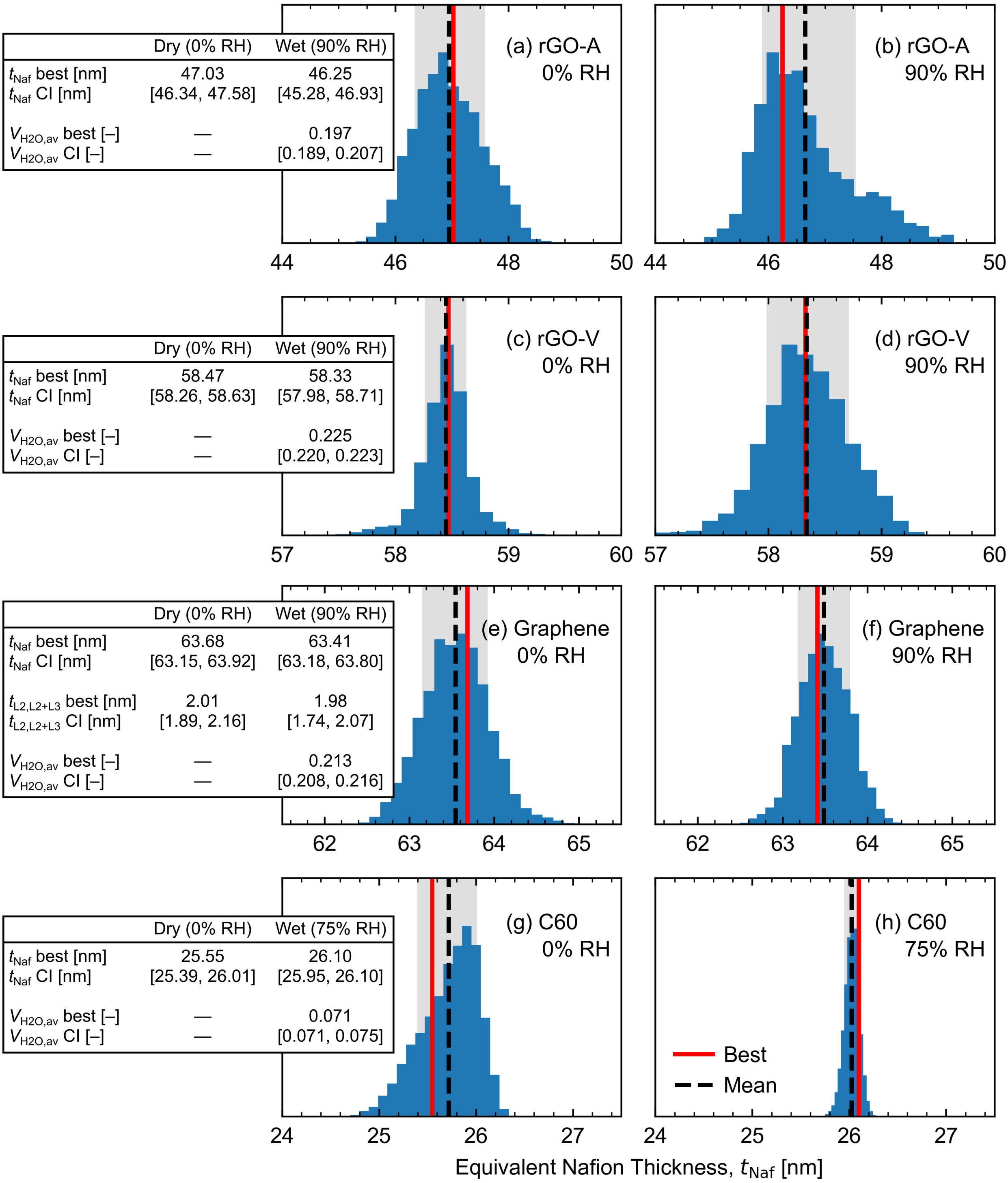

	Dry (0% RH)	Wet (90% RH)
t_{Naf} best [nm]	47.03	46.25
t_{Naf} CI [nm]	[46.34, 47.58]	[45.28, 46.93]
$V_{H2O,av}$ best [–]	—	0.197
$V_{H2O,av}$ CI [–]	—	[0.189, 0.207]

	Dry (0% RH)	Wet (90% RH)
t_{Naf} best [nm]	58.47	58.33
t_{Naf} CI [nm]	[58.26, 58.63]	[57.98, 58.71]
$V_{H2O,av}$ best [–]	—	0.225
$V_{H2O,av}$ CI [–]	—	[0.220, 0.223]

	Dry (0% RH)	Wet (90% RH)
t_{Naf} best [nm]	63.68	63.41
t_{Naf} CI [nm]	[63.15, 63.92]	[63.18, 63.80]
$t_{L2,L2+L3}$ best [nm]	2.01	1.98
$t_{L2,L2+L3}$ CI [nm]	[1.89, 2.16]	[1.74, 2.07]
$V_{H2O,av}$ best [–]	—	0.213
$V_{H2O,av}$ CI [–]	—	[0.208, 0.216]

	Dry (0% RH)	Wet (75% RH)
t_{Naf} best [nm]	25.55	26.10
t_{Naf} CI [nm]	[25.39, 26.01]	[25.95, 26.10]
$V_{H2O,av}$ best [–]	—	0.071
$V_{H2O,av}$ CI [–]	—	[0.071, 0.075]

Figure S5: Histograms of equivalent Nafion thicknesses for each sample in each testing condition. The data is generated using the population of best fits given by Refl1D. Shaded regions represent 68% confidence intervals (CIs). Tables to the left of each histogram also provide average water volume fractions ($V_{w,av}$) with CIs over all Nafion layers in each respective sample.
